# Factors associated with real-world functioning in first stages of schizophrenia disorder

**DOI:** 10.1192/j.eurpsy.2021.2128

**Published:** 2021-08-13

**Authors:** C. Martínez-Cao, A. García-Fernández, M. Valtueña-García, M.P. García-Portilla, J. Bobes

**Affiliations:** Department Of Psychiatry, University of Oviedo, Oviedo, Spain

**Keywords:** psychosis, Functioning, schizophrénia, recent onset

## Abstract

**Introduction:**

Schizophrenia is one of the most disabling diseases affecting the patient’s ability to live independently, to be socially active and to work or study^1,2^. Therefore, identifying predictors of functioning in the first stages of the disease is important to prevent a negative progression of functional outcome in these patients^3^.

**Objectives:**

To identify the factors associated with real-world functioning in patients with recent onset of the disease.

**Methods:**

Secondary analysis of a cross-sectional, naturalistic study. 84 patients with Schizophrenia (F20), aged 18-71 in their first five years of the disorder under maintenance treatment. Assessments: PANSS, CDS, CGI-S, CAINS; functioning: PSP, cognition: MATRICS. Statistical analysis: student-t test, ANOVA, Pearson correlation and lineal regression.

**Results:**

Mean age (SD): 31.30 (10.08); men: 62.8%. Statistical significant differences (p<0.05) were found in work status, benzodiazepines and antidepressants use. Furthermore, significant correlations (p<0.05) were found with depressive, positive and negative symptoms (avolition, anhedonia, alogia and affective flattening) and cognition. A significant predictive model was obtained that explains the 72.1% of the variance [F(5,74)= 20.952; p< 0.001]. This model included depressive symptoms (B= -0.940; p= 0.001), negative symptoms (B= -1.696; p< 0.001), avolition and anhedonia (B= -0.643; p= 0.001), affective flattening and alogia (B= 1.197; p= 0.003), and visual learning (B= 0.202 p= 0.039).

**Conclusions:**

Negative and depressive symptoms are the main determinants of real-world functioning in patients with recent onset of schizophrenia. Visual learning also contributes to this outcome. On the other hand, the positive relationship between expressive domain and functioning needs furthermore investigation.

**Disclosure:**

No significant relationships.

